# An in vitro bioengineered model of the human arterial neurovascular unit to study neurodegenerative diseases

**DOI:** 10.1186/s13024-020-00418-z

**Published:** 2020-11-19

**Authors:** Jerome Robert, Nicholas L. Weilinger, Li-Ping Cao, Stefano Cataldi, Emily B. Button, Sophie Stukas, Emma M. Martin, Philip Seibler, Megan Gilmour, Tara M. Caffrey, Elyn M. Rowe, Jianjia Fan, Brian MacVicar, Matthew J. Farrer, Cheryl L. Wellington

**Affiliations:** 1 Department of Pathology and Laboratory Medicine, University of British Columbia, Vancouver, British Columbia V6T 1Z3 Canada; 2 Djavad Mowafaghian Centre for Brain Health, University of British Columbia, 2215 Wesbrook Mall, Vancouver, British Columbia V6T 1Z3 Canada; 3 Institute of Clinical Chemistry, University hospital Zurich, 8000 Zurich, Wagistrasse 14, CH-8952 Schlieren, Switzerland; 4 Centre for Applied Neurogenetics, University of British Columbia, Vancouver, British Columbia V6T 1Z3 Canada; 5 Institute of Neurogenetics, University of Luebeck, 23562 Luebeck, Germany; 6 Laboratory for Neurogenetics & Neuroscience, McKnight and Fixel Institutes, University of Florida, Gainesville, 32610 USA; 7 School of Biomedical Engineering, University of British Columbia, Vancouver, British Columbia V6T 1Z3 Canada; 8 International Collaboration on Repair Discoveries, University of British Columbia, Vancouver, British Columbia V5Z 1M9 Canada

## Abstract

**Introduction:**

The neurovascular unit (NVU) – the interaction between the neurons and the cerebrovasculature – is increasingly important to interrogate through human-based experimental models. Although advanced models of cerebral capillaries have been developed in the last decade, there is currently no in vitro 3-dimensional (3D) perfusible model of the human cortical arterial NVU.

**Method:**

We used a tissue-engineering technique to develop a scaffold-directed, perfusible, 3D human NVU that is cultured in native-like flow conditions that mimics the anatomy and physiology of cortical penetrating arteries.

**Results:**

This system, composed of primary human vascular cells (endothelial cells, smooth muscle cells and astrocytes) and induced pluripotent stem cell (iPSC) derived neurons, demonstrates a physiological multilayer organization of the involved cell types. It reproduces key characteristics of cortical neurons and astrocytes and enables formation of a selective and functional endothelial barrier. We provide proof-of-principle data showing that this in vitro human arterial NVU may be suitable to study neurovascular components of neurodegenerative diseases such as Alzheimer’s disease (AD), as endogenously produced phosphorylated tau and beta-amyloid accumulate in the model over time. Finally, neuronal and glial fluid biomarkers relevant to neurodegenerative diseases are measurable in our arterial NVU model.

**Conclusion:**

This model is a suitable research tool to investigate arterial NVU functions in healthy and disease states. Further, the design of the platform allows culture under native-like flow conditions for extended periods of time and yields sufficient tissue and media for downstream immunohistochemistry and biochemistry analyses.

## Background

The brain consumes ~ 20% of total body oxygen and glucose utilization despite representing only 2% of total body mass [1, 2]. These high metabolic demands vary both temporally and spatially in the brain, and are met by the coordinated action of several cell types known collectively as the neurovascular unit (NVU) [3, 4]. Neural activity increases local cerebral blood flow (CBF) through a process known as neurovascular coupling [5]. This process links neuronal glutamate release to neuronal nitric oxide (NO) secretion that modulates vascular tone of nearby smooth-muscle cells (SMC) in the arterioles, as well as ATP-triggered astrocyte calcium waves that regulate the release of vasoactive molecules that modulate vascular tone of adjacent pericytes in the capillaries [6–9]. Endothelial cells (EC) within the NVU form the blood-brain barrier (BBB) that restricts blood-brain exchange and regulates brain waste excretion [10]. Disease-associated changes in CBF and the BBB are observed in many neurodegenerative disorders including Alzheimer’s disease (AD) [3, 11]. As such, there is tremendous interest in developing cell-based models that mimic the BBB and NVU. Such models would greatly facilitate gaining a better understanding of the interactions between neurons and the vasculature in both physiological and pathophysiological conditions. If made with human cells, they would also provide an invaluable translational platform for the development of neurotherapeutics.

Tissue engineering, organoid culture, and microfluidic technologies have emerged in the last decades as powerful research tools to study how different cell types interact in the context of their native extracellular matrices (ECM), thus driving next-generation models of human disease [12–14]. Among the many models relevant to the central nervous system developed thus far, the most advanced include: i) The Parker model that links a BBB microfluidic chip containing EC, pericytes and astrocytes to a brain microfluidic chip composed of neurons and astrocytes via artificial cerebrospinal fluid perfusion [13]. ii) The Svendsen model consisting of a single microfluidic chip where the vascular chamber of EC is separated from the brain chamber consisting of neurons, astrocytes and pericytes by a semi-permeable membrane [15]. These groups, and many others, have focused their efforts on modeling the microvasculature, given the importance of brain capillaries in neuronal function and the need for better models to assess drug uptake across the BBB.

By contrast, we aimed to develop a model of the large arterial NVU, as cerebral arteries and arterioles regulate many physiological and pathophysiological processes important for brain function [16, 17]. We recently developed a human cerebrovascular model consisting of primary EC and SMC cultured with or without astrocytes, mimicking penetrating and leptomeningeal arteries, respectively [12]. Using this model we demonstrated the possibility to study key vascular pathophysiological features of AD in vitro, namely the accumulation of beta amyloid (Aβ) in the vascular wall and subsequent vascular inflammation, which comprises cerebral amyloid angiopathy (CAA) [12, 18, 19]. However, the previously reported model lacked neurons and relied solely on exogenous recombinant Aβ, and thus, is limited in its ability to study the role of neuronal biology and neurovascular function.

In the present study, we describe expansion of this platform to generate a model of the arterial NVU composed of primary human EC, SMC and astrocytes cultured in the presence of human induced pluripotent stem cells (iPSC)-derived glutamatergic cortical neurons. Under luminal native-like flow conditions, this method creates perfusible vessels that can be sampled from both the “brain” and “blood” sides. Histological analyses confirmed a multi-layer structure similar to native human cerebrovascular tissues, and biochemical analysis confirmed the presence of a tight endothelial barrier separating a closed “brain” compartment from a separate “blood” compartment that circulates through the vessel lumen. We further showed that iPSC-derived neurons cultured in this bioengineered arterial NVU were electrically excitable and could both secrete glutamate and had measurable α-amino-3-hydroxy-5-methyl-4-isoxazolepropionic acid receptor (AMPAR) currents, suggesting possible synapse formation. Finally, we confirmed the potential to study key elements of arterial pathophysiology relevant to AD in vitro, as endogenous Aβ peptides were produced by neurons and transported from the “brain” compartment to the “blood” compartment, where they gradually accumulated in the vascular wall with greater deposition of Aβ40 than Aβ42. Endogenous phosphorylated tau was also confirmed to deposit in the vascular wall. The model described here thus serves as a controlled platform that can be used to interrogate the physiology of the human arterial NVU, including the possibility of measuring tau, neurofilament light (NF-L), glial fibrillary acidic protein (GFAP), and ubiquitin carboxyl-terminal hydrolase L1 (UCH-L1) as brain biomarkers.

## Methods

### Culture of iPSC-derived neurons

Glutamatergic cortical neurons were derived from human iPSC using a modified protocol from Shi et al [20] Briefly, iPSC (line L2131 [21]) were maintained in mTesRTM1 medium (StemCell). Seven days after the last passage, iPSC were groomed by removing any colonies having an appearance of differentiated cells, irregular borders or a transparent-center. IPSC were washed with Dulbecco’s Modified Eagle Media (DMEM)/F12 (Invitrogen), dissociated into single cells using accutase (Invitrogen) and filtered through a 0.45 μm cell strainer. After 2 washes with IPS media (4:5 DMEM/F12, 1:5 knockout serum replacement [KOSR], 15 mM HEPES, 1% glutamine, 1% MEM-non essential amino acids [NEAA], 0.1 mM β-mercaptoethanol, 10 ng/mL human fibroblast growth factor 2 [hFGF2]), iPSC were plated at a density < 200,000 cells/cm^2^ on gelatin-coated plates in IPS medium containing 10 μM ROCK-Inhibitor (Y-27632, Stemcell Technologies). After 1 h at 37 °C, non-adherent cells were collected and suspended in murine embryonic fibroblast (MEF) conditioned media containing 10 μM Y-27632 and 20 ng/mL of human FGF and plated on Matrigel® matrix (Corning)-coated plates at a density of 1–1.5 × 10^6^ per 6-well plate. MEF medium was changed daily until cells were 95% confluent, which was usually after 1 day. To initiate neuronal differentiation, 2 mL of KSR (Knockout DMEM with 15% KOSR, 1% glutamine, 1% MEM-NEAA, 0.1 mM β-mercaptoethanol) were added to the cells for 4 days. On day 5, KSR media was gradually replaced by neural maintenance medium (NMM: 1:2 DMEM/F12, 1:2 neurobasal medium, 0.25% N2 supplement, 0.25 μg/mL insulin, 0.5% MEM-NEAA, 50 μM M2-ME, 1% neuroCult SM1 Neuronal supplement, 1% glutamine, 1% Pen/strep) medium at a ratio of 3:1 KSR:NMM on day 5, 2:2 on day 7, 1:3 on day 9, and 100% NMM containing 1 μM dorsomorphine, 10 μM transforming growth factor-β (TGF-β) inhibitor SB 431542, 10 μM Y-27632 on day 11. On day 12 of differentiation, medium was removed and tissue was dissociated into clumps using a pipette. Cells were plated onto 6 cm dishes coated with poly-D-lysine/laminin in NMM. On day 13–17 of differentiation, media was removed and NMM supplemented with 20 ng/mL human FGF and 20 ng/mL of human brain derived neurotrophic factor (BDNF) was added. On day 18 of differentiation, rosettes were manually picked with a sterile pipette and plated in NMM supplemented with 20 ng/mL of BDNF, 20 ng/mL of glial derived neurotrophic factor (GDNF), and 0.2 mM ascorbic acid on poly-D-lysine/laminin-coated 6-well plates. From day 19–22 of differentiation, media was fully refreshed every other day. On day 23 of differentiation, rosettes were again manually picked with a sterile pipette and plated in NMM supplemented with 20 ng/mL of BDNF, 20 ng/mL of GDNF, and 0.2 mM ascorbic acid on poly-D-lysine/laminin-coated 6-well dishes. From day 24–27 of differentiation, medium was refreshed every other day. On day 28 of differentiation, medium was removed, cells were washed with PBS and dissociated using accutase. After 10–15 min, cells were lifted by pipetting up and down before collecting and centrifuging at 160 g for 5 min. Cells were suspended in complete NMM and plated on poly-D-lysine/laminin coated 6-well dishes at a density of 1 × 10^6^ cells per well. From day 29 of differentiation on, medium was refreshed with full NMM every 3 days. IPSC-derived neurons were genotyped as described [22] as apoEε3/4 .

### Isolation and culture of vascular cells

All experiments were conducted under an approved clinical protocol (UBC Clinical Ethics Research Board H13–02719) after obtaining written informed consent. Human umbilical vein endothelial cells (EC) and human umbilical cord myofibroblasts (SMC) were isolated as described [23]. Briefly, EC were isolated using the instillation method, where the vein lumen was filled with a solution of collagenase (2 mg/mL, Collagenase A, Roche) in serum-free DMEM (Invitrogen) before clamping both ends. After 20 min at 37 °C, Advanced DMEM (Gibco) supplemented with 1% L-glutamine, 0.05% penicillin/streptavidin (pen/strep) and 10% fetal bovine serum (FBS) (Invitrogen) was flushed through the lumen and the cell suspension was centrifuged at 1200 rpm for 5 min. EC were expanded in full endothelial growth medium (EGM™-2, LONZA Inc.,), supplemented with vascular endothelial growth factor (VEGF), human recombinant insulin-like growth factor-1 (hrIGF-1), human epidermal growth factor (hEGF), amphotericin-B, hydrocortisone, ascorbic acid, heparin, and 2% (FBS) up to passage 10 with media changed every 3–4 days. SMC were isolated by mincing the vessel wall into small pieces (~ 2–3 mm) and incubating at room temperature for 20 min without medium under sterile laminar flow to ensure physical attachment of the pieces. Advanced DMEM (Invitrogen) supplemented with 1% L-glutamine, 0.05% pen/strep and 10% FBS was subsequently added to the minced vessels and adherent cells were expanded up to passage 10 with media changed every 3–4 days. EC and SMC from different donors were genotyped as apoEε3/3 and apoEε3/4. Human primary apoEε3/3 astrocytes (Sciencell) were cultivated in astrocyte media (Sciencell) supplemented with astrocyte growth factor (Sciencell), 0.05% pen/strep and 2% FBS up to passage 5 with media changed every 3–4 days.

### Bioengineering the in vitro *arterial* NVU

Bioengineered constructs were fabricated using a dynamic, semi-pulsatile flow bioreactor system. Tubular biodegradable scaffolds (length 1.5 cm and inner diameter 2 mm) were produced as previously described [12]. Briefly, non-woven polyglycolic acid (PGA, Biomedical Structure) meshes (thickness: 1 mm and density: 70 mg/cc) were dip-coated with polycaprolactone (PCL, Sigma Aldrich) by dipping PGA mesh in a solution of 1.75% (w/w) PCL/tetrahydrofuran (THF) solution (Sigma Aldrich), shaping into tubes using heat, and externally coating with a 10% PCL/THF (w/w) solution. Scaffolds were sterilized by immersion in 70% ethanol for 30 min, followed by three PBS washes and finally immersion in advanced DMEM supplemented with 10% FBS for at least 12 h. Confluent SMC were washed with PBS, lifted from a 10 cm plate using 1 mL of trypsin (GIBCO, 5 min. 37 °C) and collected using 3 mL Advanced DMEM followed by 5 min of centrifugation at 300 g. The supernatant was removed and 2-3 × 10^6^ SMC were suspended in 15 μL of thrombin (Sigma Aldrich 100 mU/mL PBS). 15 μL of fibrinogen (Sigma Aldrich 15 fibrinogen, 10 mg clottable protein/mL in PBS) was then added to the thrombin/SMC and the mixed solution was seeded on the inner surface of the scaffold to a final density of 2–3 × 10^6^ cells/cm^2^. The seeded scaffold was incubated under static conditions in Advanced DMEM supplemented with 10% FBS, 1% L-glutamine and 0.05% pen/strep and 1.5 mM L-ascorbic acid (Sigma Aldrich). After 3 to 5 days, advanced DMEM supplemented with 10% FBS, 1% L-glutamine and 0.05% pen/strep and ascorbic acid was flowed through the lumen of the vessel using a peristaltic pump to mimic blood flow for 7 days. Confluent EC were washed with PBS, lifted from a 10 cm plate using 1 mL of trypsin (5 min at 37 °C) and collected in 3 mL of Advanced DMEM/ containing 10% FBS per plate follow by 5 min of centrifugation at 300 g. After removing the supernatant, EC were suspended in complete EGM2 containing 10% FBS at a density of 40 × 10^6^ cells/mL. Vascular intermediates were then seeded with EC to a final density of 1 × 10^6^ cells/cm^2^ on the luminal side and cultivated first in static conditions in full EGM™-2 supplemented as above. After 3 days, confluent astrocytes were washed with PBS, lifted from a 10 cm plate using 1 mL of trypsin (5 min at 37 °C) and collected in 3 mL of Advanced DMEM containing 10% FBS per plate follow by centrifugation at 300 g for 5 min. After removing the supernatant, 1 × 10^6^ astrocytes were suspended in 10 μL of thrombin (as above), then mixed with 10 μL of fibrinogen (as above) and directly seeded on the antelumen side of the tissue at a density 1 × 10^6^ cells/cm^2^. After 5 min at RT, tissue constructs were placed in complete astrocyte media under static conditions. After 24 h, confluent iPSC-derived neurons (age 60 to 80 days) were washed twice with PBS, lifted from a 10 cm plate using 1 mL of acutase (GIBCO, 10 min at 37 °C) and collected in 3 mL of completed NMM media per plate followed by centrifugation at 300 g for 5 min. The supernatant was removed and neurons were suspended in 10 μL ice cold Matrigel® matrix as a cell carrier. Neurons were seeded on the antelumen side of the engineered vessel at a density of 2 × 10^6^ cells/cm^2^. Tissues were maintained at RT for 5 min until gelation of the Matrigel® matrix was complete before mounting in the bioreactor with completed NMM media both in the tissue (with Y-27632) and circulation chamber. Tissues were maintained under flow conditions for a maximum of 21 days before experiments.

### Green fluorescent protein (GFP) electroporation

Neurons were transfected with the pmaxGFP vector (Lonza) using the Nucleofector 2b (Lonza) device. Briefly, neurons were washed twice with PBS and detached by adding accutase to the wells for 5 to 15 min. Neurons were collected in NMM+ as described above and centrifuged at 250 g for 3 min. They were then suspended in Mouse NSC Nucleofector Solution (Lonza) at a density of 4 × 10^6^ neurons/100 μL with 4 μg of pmaxGFP, followed by transfection in the Nucleofector 2b using the program B-016. 500 μL warm NMM+ was then directly added to the transfected cells. After 5 min, cells were centrifuged at 250 x g at room temperature for 3 min, suspended in 10 μL Matrigel® matrix and 10 μL NMM with Y-27632 and seeded on the antelumen of the tissues as above.

### Electrophysiology

Bioengineered tissues were carefully cut longitudinally in thirds, and transferred to a recording chamber continually perfused (1–2 mL/min) with artificial cerebral spinal fluid (aCSF) consisting of: 126 nM NaCl, 26 mM NaHCO_3_, 2.5 mM KCl, 1.25 mM NaH_2_PO_4_, 2 mM MgCl_2_, 2 mM CaCl_2_, and 10 mM glucose. aCSF was continuously bubbled with 95% O_2_/5% CO_2_ and warmed to 33 °C using a stage heater (Luigs & Neumann). Whole-cell patch clamp recordings were obtained using thin-walled borosilicate glass microelectrodes (Warner) pulled to a tip resistance of 3–5 MΩ with a P-97 Flaming/Brown Micropipette Puller (Sutter Instruments). Electrodes were filled with an intracellular recording solution containing: 108 mM K-gluconate, 3 mM KCl, 2 mM MgCl_2_, 8 mM Na-gluconate, 1 mM K_2_-EGTA, 230 μM CaCl_2_, 50 μM Alexa-594, 4 mM K_2_-ATP, 200 μM Rhod-2 tripotassium salt (ThermoFisher), and 300 μM Na_3_-GTP at pH 7.25 with 10 mM HEPES. Recordings were made using a MultiClamp 700B amplifier and a Digidata 1440A digitizer (Axon Instruments, Molecular Devices) controlled via Clampex 10.7 acquisition software. Cells were voltage clamped at − 60 mV for glutamate puff experiments and passively current clamped (i.e. passive membrane potential monitoring) for Ca^2+^-imaging experiments. Stimulation trains (200 pA, 5 ms/pulse, 20 Hz, 5 s total) were applied for transient membrane depolarizations to trigger Ca^2+^ entry. Access resistance was always < 20 MΩ. Glutamate (200 μM) was transiently applied (5 s puffs) by using a puff electrode connected to a Picospritzer II (General Valve Corporation). The relative magnitude of AMPAR currents were quantified as normalized charge (i.e. the area under the curve) to control for the variability of the peak current responses. 6-cyano-7-nitroquinoxaline-2,3-dione (CNQX; 10 μM) was bath applied and was purchased from Tocris.

### Two-photon microscopy

All experiments were performed on a LSM MP710 2-photon imaging system (Zeiss). Cells were identified for whole-cell patch clamp and imaging using either widefield infrared illumination captured with a DAGE IR-1000 camera (DAGE-MTI). This was preferable to patching GFP-labelled cells due to the sparse labeling and ease in identifying healthy neurons with transmitted illumination. GFP and/or Rhod-2 imaging was achieved by 2-photon excitation with a Ti:Sapphire Chameleon Ultra II 2-photon laser (Coherent) tuned to 850 nm. Images were acquired with a Zeiss 20X-W/1.0 NA objective at a pixel resolution of either 512 × 512 or 256 × 256 for fast Rhod-2 Ca^2+^-imaging. Emission light was split with a 575 nm longpass filter, and green and red emissions were filtered with 535/50 nm and 630/75 nm bandpass filters, respectively (all from Chroma Tech). Emission light was collected with LSM BiG GaAsP detectors from Zeiss, and data were acquired using Zen software (Zeiss) and analyzed in Fiji.

### Glutamate quantification

Media was removed and cultures received a treatment of 56 mM KCl or regular Hanks Buffered Salt Solution (HBSS) for 30 min. Glutamate was measured by high-pressure liquid chromatography (HPLC) coupled to electrochemical detection (ALEXYS Neurotransmitter platform, Antec). 5 μL of sample was automatically injected (AS 110 Autosampler, Antec) onto an Acquity UPLC HSS T3 analytical column (1 mm inner diameter, 50 mm length; Waters) perfused at a flow rate of 200 μL/min (LC 110S pump, Antec) with a mobile phase containing 50 mM phosphoric acid, 50 mM citric acid, 0.1 mM EDTA, and 2% acetonitrile (pH 3.5). At the end of each sample, a solution of 50 mM phosphoric acid, 50 mM citric acid, 0.1 mM EDTA, and 50% acetonitrile (pH 3.5) was run to flush the column before the next sample. Each sample was mixed with a solution of 0.025 g of ortho-phthalaldehyde (a derivatization agent) in 250 μL of methanol, 250 μL of 1 M sodium sulfite, and 4.5 mL of 0.1 M borate buffer (pH 10.4), for analytic detection. Glutamate was detected by means of an electrochemical detector (Decade II, Antec) with the cell potential set at 0.85 V vs. salt bridge. Retention times were 3.1 ± 0.4 min.

### ApoE measurement

Secreted apoE levels in both the tissue chamber and circulation media were quantified by an apoE ELISA protocol as described previously [24]. 1 μM of the liver-X-receptor agonist GW3965 or dimethyl sulfoxide (DMSO) vehicle control were circulated through the lumen for 96 h before collecting media. Fluorescence was read at 325_Ex_/420_Em_ on an Infinite® Tecan M200 Pro plate reader (Tecan Life Science).

### Histology and immunohistochemistry

Bioengineered arterial NVU were prepared for cryopreservation by being washed in PBS, cut in 5 mm cross-sectional pieces and fixed in 4% paraformaldehyde (PFA, Sigma Aldrich). After 30 min, tissues were washed three times in PBS, cryopreserved in 20% sucrose PBS solution for a minimum of 60 min, cut in half longitudinally, embedded in 5% bovine skin gelatin (Sigma Aldrich) and 20% sucrose in PBS and stored at − 80 °C until further processed. Samples were processed on a cryotome (chamber − 30 °C and object − 25 °C) to generate 20 μm sections that represented a longitudinal cross-section of the arterial NVU. Samples were stored at − 80 °C until analysis. De-identified human AD brain tissues (cortex Brodmann area 9) were received from the Harvard Brain Tissue Resource Center under UBC Clinical Research Ethics Board protocol C04–0595 and cut into 20 μm sections using a cryotome. On the day of immunohistochemistry, sections were rehydrated in PBS for 2 × 10 min before blocking for 30 min in 5% goat serum and 1% BSA in PBS. For immunohistochemistry on fresh tissues, bioengineered arterial NVU were washed with PBS and fixed in 4% PFA. After fixation, tissues were washed three times with PBS, and were cut in half longitudinally, mounted with the abluminal side facing upward (lumen facing the microscopy slide) and directly processed for staining. After staining (see below), tissues were covered with a coverslip to specifically image the antelumen.

For immunofluorescence, cryopreserved sections (arterial NVU and human brain sections) and fresh arterial NVU were blocked in 5% donkey serum and 1% BSA in PBS for 30 min at RT, incubated overnight at 4 °C with specific antibodies against the endothelial markers platelet endothelial cell adhesion molecule (CD31, RRID: AB_31432, WM59 Biolegend, 1:50) and von Willebrand factor (vWF, RRID:AB_259543, SigmaAldrich, 1:200), the SMC marker α-SM-actin (αSMA RRID:AB_476856, 1A4 SigmaAldrich, 1:200), the astrocyte markers glial fibrillary acidic protein (GFAP, RRID: AB_880203, Abcam, 1:200), and aquaporin 4 (AQ4, RRDI:AB_2039734, Alomone Labs, 1:100), the neuronal markers microtubule-associated protein 2 (MAP 2, RRID:AB_776174, Abcam, 1:200), β-tubulin III ( β-tub III, RRID:AB_2256751, Tuj1, 1:200), synapsin I (Syn, RRID:AB_2200097, Abcam, 1:200), Aβ markers Aβ 1–16 (6E10, RRID: AB_2565328, ThermoFisher Scientific, 1:50) and Aβ fibrils (OC fibril, RRID: AB_1977024, EMD Millipore, AB2286, 1:200) and phospho-Tau (AT8, RRID:AB_223648, ThermoFisher, 1:250). After three additional PBS washes, sections and fresh arterial NVU were incubated for 45 min at RT with anti-rabbit or anti-mouse Alexa-488 or Alex-594 secondary antibodies (Invitrogen). Finally, sections were washed three times in PBS and mounted in Prolong Diamond antifade containing DAPI (Invitrogen). Sections were imaged with an Axioscan inverted microscope (Zeiss) and fresh arterial NVU were imaged with an Axioscan inverted confocal microscope (Zeiss). Cryopreserved sections were stained for Haematoxylin and Eosin (Sigma Aldrich) following manufacture’s instructions.

### Aβ quantification

Luminal media was collected from the circulation loop and abluminal media was collected from the tissue chamber. For tissue biochemistry, 5 mm cross-sectional rings of tissue were crushed using a manual pestle and lysed in 150 μL radioimmunoprecipitation assay (RIPA) buffer (10 mM Tris pH 7.4, 150 mM NaCl, 1.0% NP-40, 1.0% sodium deoxycholate, 0.1% SDS and cOmplete protease inhibitor with EDTA (Roche)). After homogenization, tissue samples were centrifuged for 15 min at 14000 g at 4 °C and the RIPA soluble fraction was transferred to a new tube. 250 μL of 5 mM of guanidine (GuHCl, Sigma Aldrich) was added to the tissue pellet and incubated overnight at RT under constant agitation before centrifugation at 14000 g at 4 °C for 15 min. RIPA soluble and GuHCl soluble fractions were stored at − 20 °C until quantification. RIPA (soluble), GuHCl (insoluble) and media fractions were quantified without dilution using Aβ40 (KHB3442, Life Tech) and Aβ42 (KHB3482, Life Tech) commercial ELISA kits. Aβ levels in RIPA and GuHCL fractions were normalized to total protein concentration as measured by bicinchoninic acid (BCA) assay (Fisher) and Aβ levels in abluminal media were normalized to tissue chamber media volumes.

### Single molecule array for biomarker quantification

Total tau, GFAP, neurofilament light (NF-L) and ubiquitin carboxyl-terminal hydrolase L1 (UCH-L1) were quantified in media from the tissue chamber (abluminal) and from the circulation loop using the Neurology 4-plex A assay (Quanterix,) using the Simoa HD-1 analyzer (Quanterix) following the manufacturer’s guidelines. Abluminal and circulating media were diluted off-board 2500 and 20 fold, respectively.

### SDS-PAGE and Immunoblotting

Tissues composed of EC, SMC and astrocytes without or with neurons were lysed in RIPA buffer with Phosphostop (Roche). After 20 min on ice, tissues were crushed using a manual pestle before centrifuging for 10 min at 12000 g at 4 °C. Total protein was quantified using BCA assay. Equal amounts of total protein (25 μg) were separated by 10% acrylamide SDS-PAGE, followed by electrophoretic transfer to polyvinylidene fluoride (PVDF) membranes (Millipore). Membranes were blocked for 1 h using 1% BSA in Tris-buffered saline (TBS, 20 mM TrisBase and 150 mM NaCl, pH 7.5) containing 0.5% Triton X (TBST). Phosphorylated tau AT8 (RRID:AB_223648, ThermoFisher, 1:1000), CP13 (RRID:AB_2314223, kindly gifted by Dr. Peter Davies at Litwin-Zucker Research Center for The Study of Alzheimer’s Disease and Memory Disorders, 1:1000) and PHF1 (RRID:AB_2315150, Dr. Peter Davies, 1:1000) and total tau DA9 (RRID:AB_2716723, Dr. Peter Davies, 1:1000) were immunodetected by incubating overnight in blocking buffer at 4 °C. Membranes were washed extensively with TBST and incubated with anti-mouse, (1:1000, Jackson ImmunoResearch) secondary antibody in blocking buffer. After 1 h, membranes were washed extensively with TBST, developed using enhanced chemiluminescence (ECL, Amersham) and imaged using ChemiDoc MP imager (Biorad). Band densitometry was quantified using Fiji.

### Endothelium integrity

Restriction of paracellular transport was determined by measuring 4 kDa FITC-dextran extravasation from the lumen to the tissue chamber as described [12]. Briefly 250 μg/ml of 4 kDa FITC-dextran (Sigma Aldrich) was circulated through the bioengineered tissue lumen. After 2 h tissue media was collected and fluorescence was measured at RT on Infinite® Tecan M200 Pro plate reader (492_Ex_/518_Em_). The permeability coefficient (P_app_, cm/s) was calculated using the following equation: P_app_ = (dQ/dt)*(1/A*C_0_*60) where dQ/dt is the amount of FITC-dextran transported per minute (ng/min), A is the surface area of the tissue in cm, C_0_ is the initial concentration of FITC-dextran (ng/ml) and 60 is the conversion from minutes to seconds.

### Statistics

Comparisons between groups were performed using unpaired or paired Student t-test, or one-way ANOVA with Dunnett’s or Sidack’s multicomparison test. Dependence analyses were assessed through Pearson correlation analysis. Values below the detection limit of the ELISAs were considered as 0 for statistical analysis and plotted as gray points. Data were obtained from at least three independently seeded bioengineered arterial NVU and graphically represented as scatter or before-after plots with mean ± standard error of the mean (SEM). *P*-values of < 0.05 were considered statistically significant. All statistical analyses were performed using GraphPad Prism-5 or SPSS software.

## Results

### Production and anatomical characterization of bioengineered arterial NVU

**Fig. 1:**
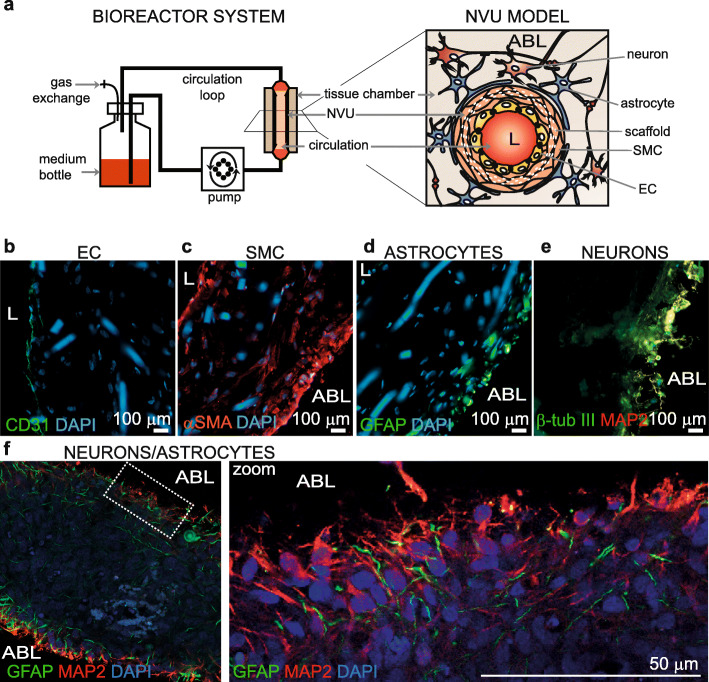
Histological structure of bioengineered arterial NVU. **a** Schematic representation of the bioreactor and arterial NVU model. **b-e** Cryopreserved bioengineered arterial NVU were cut longitudinally to show a cross-section of the NVU wall. The expression of CD31 (**b**) confirmed the presence of an endothelial cell monolayer on the luminal side of the bioengineered NVU and αSMA (**c**) confirmed the smooth muscle phenotype of the cells in the inner layer. GFAP (**d**) and MAP 2/β-tubIII (**e**) positive staining confirmed respectively the astrocyte and neuron phenotype of the cells on the out layers in radial section of the NVU. **f** Bioengineered arterial NVU were stained without cryopreservation and were mounted directly on microscopy slide with the antelumen facing the coverslip. An optical sectioning was performed using confocal microscopy to image a focal plan . Staining against GFAP and MAP 2 confirmed the imbrication of the astrocytes and neurons. L = lumen, ABL = albumen

Human arterial NVU were fabricated by sequentially seeding, in order, primary human SMC, primary human EC and primary human astrocytes into a tubular porous scaffold consisting of polyglycolic acid (PGA), and polycaprolactone (PCL), measuring 15 mm long and 2 mm in diameter as described previously [12]. 24 h after seeding astrocytes, iPSC-derived neurons (aged 60 to 80 days) were seeded on the antelumen of the tissues using Matrigel® matrix as a cell carrier. A schematic of the finished bioengineered arterial NVU and bioreactor system is provided in Fig. 1a and histology of a post-mortem endogenous human arterial NVU is provided in Sup. Fig 1. Specifically, after 3 weeks in culture under native-like luminal flow conditions, cryosections from bioengineered arterial NVU were prepared along the longitudinal axis showing a cross section of the bioengineered vessel wall and processed for immunofluorescence staining against the EC marker CD31, the SMC marker αSMA, and the astrocyte marker GFAP. We observed a multilayer tissue organization resembling a cerebral artery; with a single layer of EC on the lumen, multiple layers of SMC surrounding the endothelium and astrocyte layers on the antelumen (Fig. 1b-d). Immunofluorescence staining also demonstrated the presence of the neuronal marker β-tubulin-III and MAP 2 positive cells in the most abluminal layers of the bioengineered vessels, supporting the presence of neurons in the culture (Fig. 1e). The abluminal structure was further characterized by immunofluorescence staining against GFAP and MAP 2 by optical sectioning to image a focal plane in the abluminal side of a fresh bioengineered arterial NVU using confocal microscopy. Co-staining demonstrated that neurons and astrocytes form an imbricated network of cells on the last abluminal layers of cells with astrocytes penetrating farther into the tissue than neurons, whereas cells deeper in the tissue (~ 50 μm) were negative for both markers (Fig. 1f). Interestingly, contrary to cells grown in 2D culture, iPSC-derived neurons seeded on the bioengineered vessels do not form colonies but rather appear to be uniformly dispersed on the abluminal surface along astrocytes. Finally, H&E staining of longitudinally cut cryosections showed formation of a multilayered tissue with remaining scaffold (arrows) in the core of the vascular wall (brownish features) and the formation of extremely dense layers on the albumen (Sup Fig. 2a).

### Cellular functionality in the bioengineered arterial NVU

**Fig. 2:**
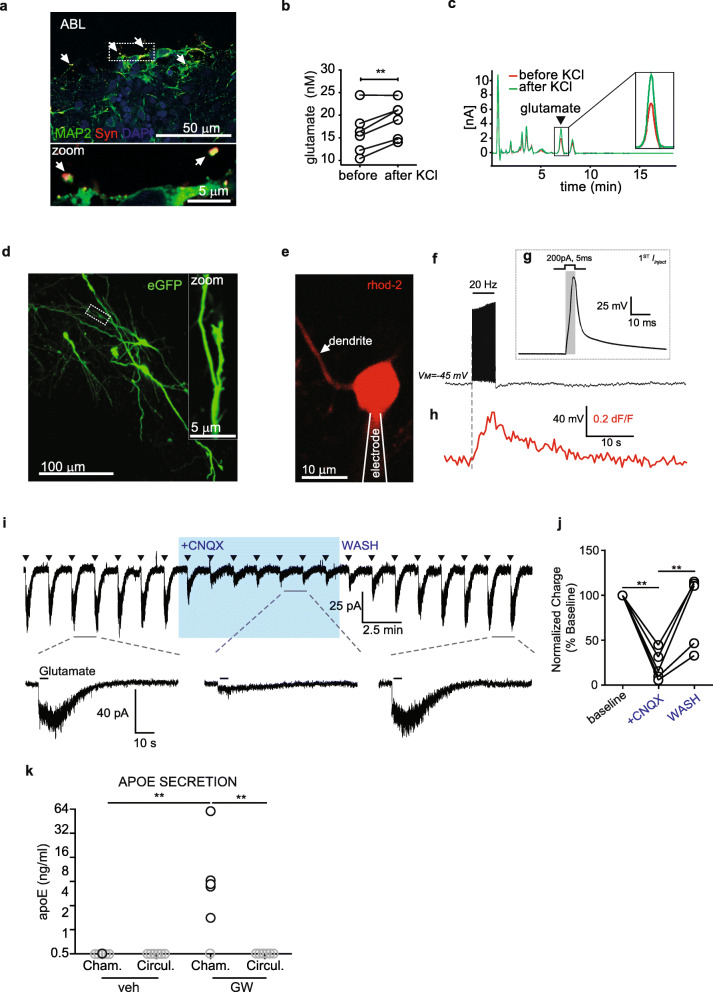
Depolarization- and glutamate-driven activity in abluminal cells indicates a neuronal phenotype and apoE secretion in the tissue chamber indicates astrocyte function and endothelial barrier formation. **a** Immunostaining against MAP 2 and synapsin-I (Syn) confirmed the presence of synapses in iPSC-derived neurons cultured in arterial NVU. **b** Glutamate release measured by HPLC showing increase after KCl treatment. **c** Example HPLC curves. **d** Two-photon Z-projection image of iPSC cells expressing eGFP. Dotted box displays region of zoomed inset, highlighting dendritic morphology and synaptic structure. **e** Example image of a whole-cell patch clamped iPSC-derived neuron dialyzed with the red Ca^2+^-indicator Rhod-2. Proximal dendrites were imaged for depolarization-induced Ca^2+^-entry. **f** Representative current-clamp trace from patched cell in ‘**e**’ during 20 Hz spike train stimulation. **g** Single current injection (200 pA, 5 ms) example from ‘**f**’ showing change in membrane potential. **h** Time-correlated Rhod-2 signal from trace ‘**e**’ showing depolarization-induced Ca^2+^-increase. **i** (Top trace) Full-length voltage-clamp recording showing glutamate puff-evoked (triangles) AMPAR currents that were amenable to block by CNQX (10 μM) and recovered in washout. (Bottom trace) Example AMPAR currents before, during, and after CNQX application. **j** Quantitative summary of normalized charge for glutamate inward currents in the presence of CNQX (*n* = 5, **P < 0.01). **k** Astrocyte and endothelium barrier functions were confirmed by treating tissues with 1 μM LXR agonist GW3965 for 96 h and measuring the levels of astrocyte-derived apoE secreted into the tissue chamber and circulation media. Values below the detection of the ELISA are plotted in gray. Points in graphed data represent individual bioengineered vessels, bars represent mean, error bars represent ±SEM and analysed by one way ANOVA **P < 0.01

Several approaches were used to evaluate neuronal function in the bioengineered arterial NUV. We first analysed synapsin-I expression using immunofluorescent staining of fresh arterial NVU. Confocal imaging confirmed punctate synapsin staining in MAP 2 positive cells (Fig. 2a, white arrows). Second, we measured glutamate release after KCl treatment using high performance liquid chromatography (HPLC) with electrochemical detection. After 3 weeks under flow conditions, tissues were treated with 56 mM KCl. After 30 min, five of the six independent tissues with neurons tested demonstrated significant increased glutamate release (Fig. 2b-c). Next, we used two-photon microscopy and electrophysiology to assess the morphology and electrical properties of the neurons and to determine their sensitivity to glutamate stimulation. Neurons were identified by sparse (1/5) green fluorescent protein (GFP) labelling, and qualitative morphological analysis by two-photon microscopy revealed a typical neuronal phenotype with long processes extending from the soma (Fig. 2d). To assess the intrinsic properties of these cells, we first tested for depolarization-induced Ca^2+^-entry by driving action potential firing. Neurons were whole-cell patch loaded with the membrane impermeant Ca^2+^-sensor Rhod-2 for Ca^2+^ imaging with 2-photon microscopy (Fig. 2e). Stimulation trains (200 pA, 5 ms/pulse, 20 Hz, 5 s total) triggered action potentials (Fig. 2f-g) and temporally correlated with Ca^2+^ entry in the soma and dendrites as measured by an increase in Rhod-2 fluorescence (Fig. 2h), suggesting that the cells were both electrically excitable and expressed voltage-gated Ca^2+^ channels. Lastly, we tested for functional expression of AMPA receptors in the membrane by exogenous glutamate stimulation. Glutamate was locally applied via a puff electrode (200 μM, 5 s) and elicited reliable inward currents in cells voltage clamped at *Vm* = − 60 mV. These currents were reversibly inhibited by bath application of the AMPA receptor antagonist CNQX (10 μM), confirming that these currents were mediated by AMPA receptor opening (Fig. 2i-j). We further characterized astrocyte function by measuring apoE secretion after stimulation with the brain-penetrant Liver-X-Receptor (LXR) agonist GW3965 added to the circulating media before collecting tissue chamber and circulation media. After 96 h, ELISA quantification confirmed a significant increase of secreted apoE in the chamber media of GW3965-treated tissues while the concentration of apoE in the circulation media was below the detection limit of the ELISA, and not different than control media (Fig. 2k), suggesting a lack of apoE transport across the endothelium in this model. These observations support astrocyte functionality via apoE secretion and a tight endothelium barrier as apoE does not cross the BBB in vivo [25]. To further assess astrocytes we immunostained cryopreserved longitudinal sections against aquaporin (AQ)4 and GFAP. AQ4 was localized in GFAP positive cells (yellow arrow) but also in GFAP negative cells (white arrow) similar to its localization patterns in human brain tissue (Sup. Fig. 2b). Finally, we assessed endothelial integrity by circulating 4 kDa FITC-Dextran through the lumen of the bioengineered arterial NVU and measured a permeability of 8.3 10E^− 9^ ± 6.06 E^− 9^ cm/s (Sup. Fig 2c). Together, these data confirm that our bioengineered arterial NVU possesses both structural and functional characteristics of native neurons and astrocytes surrounding an arterial blood vessel with a tight endothelial barrier.

#### Proof-of-principle for the utility of this bioengineered arterial NVU model to study neurodegenerative diseases

**Fig. 3:**
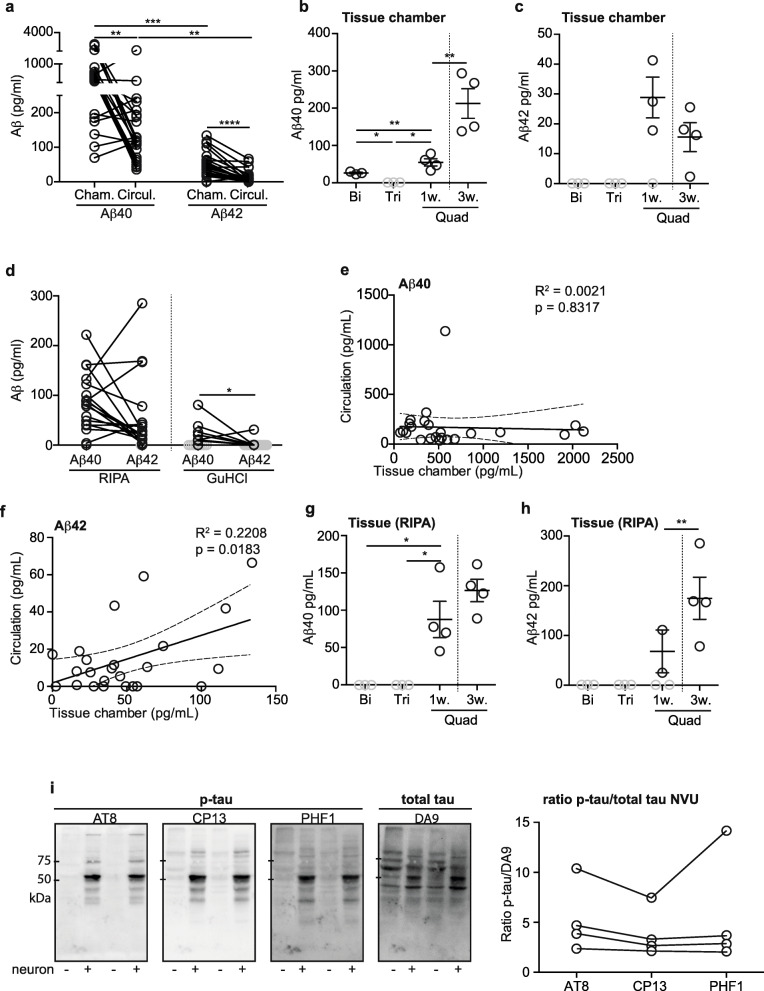
Neurons secrete Aβ that then accumulates within the vascular wall. **a** Aβ40 and Aβ42 levels were quantified by ELISA in the chamber and circulation media of bioengineered arterial NVU after 3 weeks. Aβ40 (**b**) and Aβ42 (**c**) levels in chamber media of tissues composed of EC and SCM (bipartite), EC, SMC and astrocyte (tripartite) and EC, SMC, astrocytes and neurons (NVU) after one or three weeks in culture. **d** Vascular Aβ40 and Aβ42 level in RIPA and GuHCl soluble fractions were quantified by ELISA in NVU after three weeks in culture. The correlation between the level of Aβ40 (**e**) and Aβ42 (**f**) in circulation and tissue chamber were assessed through Pearson correlation analysis. The correlation coefficient (*R*^2^) and *p*-value are shown in each panel. Aβ40 (**g**) and Aβ42 (**h**) vascular deposition were quantified in RIPA soluble fraction after a week (bipartite, tripartite and NVU) and three weeks (NVU) in culture. **i** The level of p-tau (AT8, CP13 and PHF1) was measured by Western blot in NVU and tripartite tissues and compared to total tau (DA9). Points in graphed data represent individual bioengineered vessels, bars represent mean, error bars represent ±SEM and analysed by one way ANOVA or Pearson correlation. Values below the detection of the ELISA are plotted in gray. * = *p* < 0.05, ** = *p* < 0.01, *** = *p* < 0.001

In vitro models are used as research tools to address specific physiological or pathological questions that are not feasible to study in human subjects or are difficult to translate from animal models. Here we tested the hypothesis that our bioengineered arterial NVU could be used to study cerebrovascular contributions to AD or other neurodegenerative diseases. AD is the leading cause of dementia affecting over 50 million people worldwide with a global economic burden of over one trillion USD [26]. While extracellular plaques composed of beta-amyloid (Aβ) peptides, and intracellular neurofibrillary tangles are the classical neuropathological hallmarks of AD, 90% of AD patients also have some form of cerebral vessel disease, including vascular Aβ deposition known as CAA [27]. We first used ELISA to quantify endogenous Aβ secreted from iPSC neurons in the bioengineered arterial NVU in the tissue chamber and in the circulation media as well as in tissue lysates. We found Aβ40 levels to be significantly higher than Aβ42 levels in both the tissue chamber and circulating media (Fig. 3a). Specifically, Aβ42/Aβ40 ratios were 0.078 and 0.0867 in the tissue chamber and circulating media, respectively, similar to the reported Aβ42/Aβ40 ratio in human CSF [28] and blood [29]. Notably, Aβ levels were higher in the tissue chamber than in the circulation media 4 days after the last media change (Fig. 3a), confirming that the tight endothelial barrier prevented diffusion of Aβ between the two media compartments. Because Aβ has been reported to be secreted by various cell types in vivo including EC [30], we confirmed that Aβ was predominantly secreted by neurons in our model by comparing tissues fabricated with and without neurons. ELISA quantification revealed that tissues with neurons had twice as much Aβ40 in the tissue chamber than tissues composed only of EC and SMC (bipartite, 26.42 pg/mL) and tissues composed of EC, SMC and astrocytes (tripartite, below detection limit of the ELISA, < 6 pg/mL). Further, Aβ40 concentration increased over time from 54.78 to 212.6 pg/mL after 1 week and 3 weeks in culture, respectively (Fig. 3b). Aβ42 was only detectable in tissues with neurons, as Aβ42 was below the detection limit of the ELISA (10 pg/mL) in bipartite and tripartite tissues lacking neurons. In tissue chamber media of bioengineered arterial NVU, Aβ42 levels were 28.85 pg/mL after 1 week (one sample below detection limit) and 15.56 pg/mL after 3 weeks in culture, respectively (Fig. 3c). In the circulation media, Aβ40 and Aβ42 were only detectable in tissues with neurons compared to tissues lacking neurons, where Aβ40 and Aβ42 levels were below the detection limit of the ELISAs (Sup. Fig 3a-b).

We next analyzed Aβ deposition within the arterial NVU in RIPA, corresponding to interstitial and cellular fractions, and GuHCl-soluble fractions, corresponding to the fraction containing insoluble Aβ fibrils. We confirmed the presence of Aβ40 in RIPA fraction in all 18 tissues tested, and Aβ42 in 17 of the 18 tissues with most but not all RIPA lysates containing more Aβ40 than Aβ42 (Fig. 3d). Similarly, Aβ40 levels were significantly higher than Aβ42 in GuHCL-soluble lysates with 9 tissues (50% of the total tissues analyzed) positive for Aβ40 and only 2 tissues (11%) above the ELISA detection limit for Αβ42 (Fig. 3d). These results suggest that Aβ40 accumulates to a greater extent than Aβ42 in this model, which could be explained either by higher secretion of Aβ40 as suggested in Fig. 3a, or by increased retention of Aβ40 in the tissue. To test this hypothesis, we determined whether the level of Aβ in the circulation media correlated with the level in the tissue chamber, reasoning that if increased Aβ40 secretion causes the enhanced Aβ40 accumulation, Aβ40 and Aβ42 levels in the tissue chamber and circulation media should correlate. However, if Aβ40 is more readily retained in the tissue, Aβ42 levels in the tissue chamber and circulation media should correlate but Aβ40 levels should not. While Aβ40 levels in the circulation media were independent of those in the tissue chamber (*R*^2^ = 0.00218, *p* = 0.8317), Aβ42 levels in the circulating media and tissue chamber significantly correlated (*R*^2^ = 0.2208, *p* = 0.0183), (Fig. 3e-f), suggesting that Aβ40 preferentially accumulates in the NVU. We further confirmed that the origin of accumulated vascular Aβ was neuronal by comparing tissues with and without neurons. While Aβ40 and Aβ42 tissue concentrations increased over time in tissues with neurons (1 vs 3 week), Aβ40 and Aβ42 levels in tissues lacking neurons were below the detection limit of the ELISAs (Fig. 3g-h). We further investigated Aβ tissue localization by immunostaining cryopreserved longitudinal tissue sections against Aβ fibrils (OC-fibril) or against Aβ 1–16 (6E10) in comparison to human brain samples. Staining against OC-fibril showed extracellular deposition within the β-tub III (neuron marker) positive layers similar to the signal in human brain (white arrow), but also deeper within the tissue around the remaining of the scaffold (blue arrow) (Sup. Fig. 4a). Staining against 6E10 showed both extracellular signal (white arrow) and co-localization with the neuron marker MAP 2 (yellow arrow) like observed in human brain (Sup. Fig. 4b).

We also used immunoblotting to test if endogenous phosphorylated tau (p-tau), the first constituent of neurofibrillary tangles, was detectable in our bioengineered arterial NVU. We confirmed the presence of p-tau in tissues with neurons using two different p-tau antibodies CP13 (pSer202) and PHF1 (pSer396/Ser404) as well as the early tangle marker AT8 (pSer202/Thr205) in comparison to a marker of total tau (DA9). As expected, tissues lacking neurons were negative for AT8, CP13 and PHF1, strongly supporting lack of p-tau (Fig. 3i). We also observed many cross-reactive bands using the total tau marker DA9 with distinct patterns in tissues with and without neurons. The p-tau/total tau ratio was calculated and graphed to show bioengineered arterial NVU variation (Fig. 3i). Finally, we immunostained longitudinally cut cryopreserved tissues against phospho-tau (AT8) and the neuronal marker MAP 2. Staining against AT8 showed both neuronal localization (yellow arrow) but also extracellular diffuse deposition (white arrow) similar to the signal observed in human brain (Sup. Fig. 3c). In addition, we observed a signal associated with the remaining scaffold (blue arrow) suggesting unspecific binding of AT8 to PGA or PCL. Taken together, these results support the potential utility of this model to study mechanisms related to vascular Aβ and tau deposition.

#### Characterization of NF-L, GFAP, total tau and UCH-L1 biomarkers in the engineered NVU

**Fig. 4:**
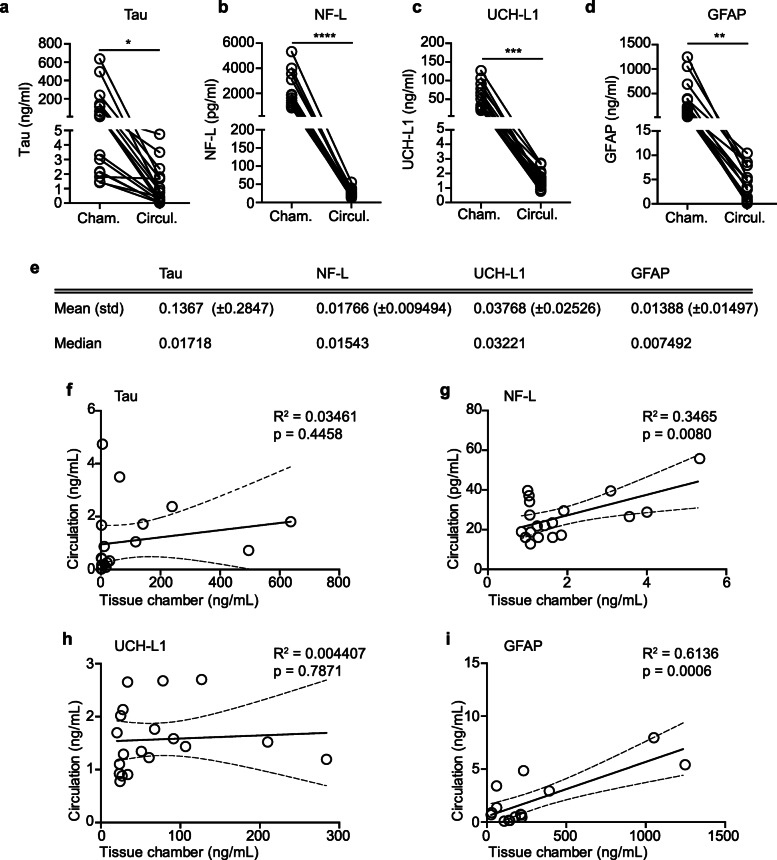
Fluid biomarkers levels in tissue chamber vs. circulation. Total tau (**a**) NF-L (**b**) UCH-L1 (**c**) and GFAP (**d**) were quantified in tissue chamber and circulation media four days after last medium change. **e**) Ratio circulation:chamber calculation. The correlation between the level of total tau (**f**) NF-L (**i**) UCH-L1 (**j**) and GFAP (**k**) in circulation and tissue chamber were assessed through Pearson correlation analysis. Points in graphed data represent individual bioengineered vessels, bars represent mean, error bars represent ±SEM and analysed by paired Student’s t-test or Pearson correlation. The correlation coefficient (*R*^2^) and *p*-value are shown in each panel. * = *p* < 0.05, ** = *p* < 0.01, *** = *p* < 0.001, **** < *p* = 0.0001

**Fig. 5:**
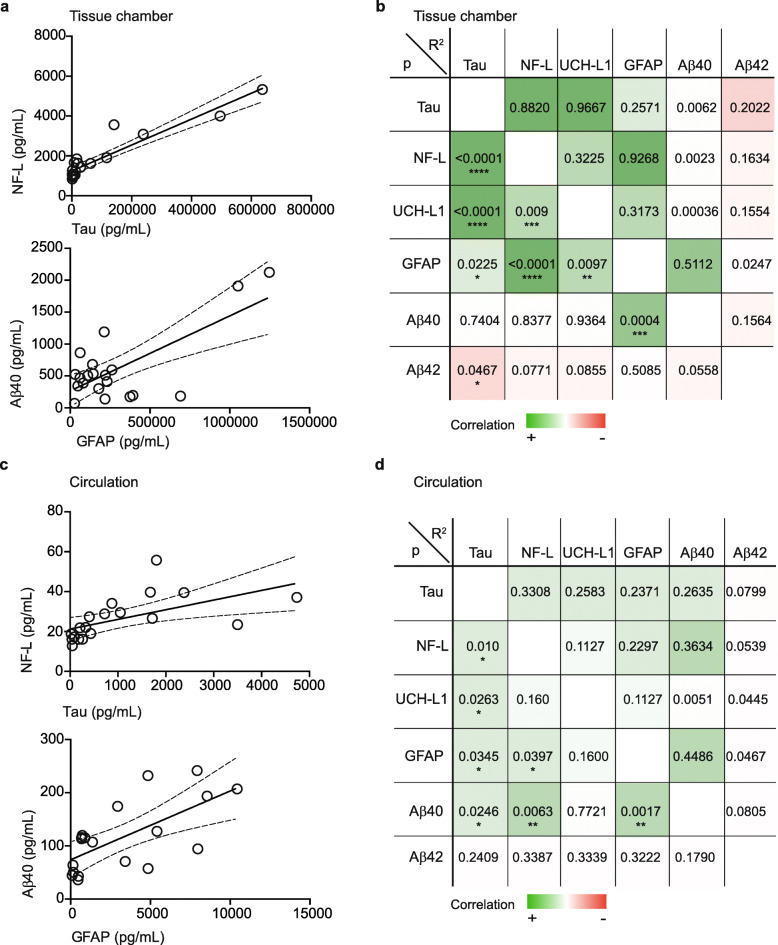
Fluid biomarkers and Aβ levels dependence in tissue chamber and circulation. The correlation between the level of total tau, NF-L, UCH-L1, GFAP, Aβ40 and Aβ42 in tissue chamber (**a-b**) and circulation (**c-d)** were assessed through Pearson correlation analysis. The correlation coefficient (*R*^2^) and *p*-value are displayed and significant correlations are graphed. * = *p* < 0.05, ** = *p* < 0.01, *** = *p* < 0.001, **** < *p* = 0.0001

The possibility to study fluid biomarkers using a human-based in vitro model of the cerebrovasculature has, to our knowledge, not yet been investigated. Nevertheless, a biofidelic human model would have undeniable translational advantages over animal models to validate or discover novel biomarkers of disease. As a proof-of-principle, we tested whether total tau, neurofilament light (NF-L), ubiquitin carboxy-terminal hydrolase L1 (UCH-L1) and glial fibrillary acidic protein (GFAP) could be quantified in both chamber and circulation media using an ultrasensitive single molecule immunoassay (Simoa). After 3 weeks in culture and 4 days after the last media change in the circulation loop, we observed that the levels of all four biomarkers were significantly higher in the tissue chamber compared to the circulation media (Fig. 4a-d). These results were confirmed by calculating the circulation:chamber ratio (Fig. 4e), providing additional support for a tight endothelial barrier in our model. Interestingly, we confirmed a positive correlation between chamber and circulation media for both NF-L and GFAP but no correlation for total tau and UCH-L1 (Fig. 4f-i). Finally, we used correlation analyses to compare the level of each biomarker in the tissue chamber and circulation media. For this, we selected two pairs of biomarkers for dependence analysis, namely NF-L:total tau and GFAP:Aβ40 for which we provide the plotted data (Fig. 5a and c) and a summary table for all other analyses (Fig. 5b and d). Dependence analyses show that levels of total tau, NF-L, GFAP and UCH-L1 strongly and positively correlate with each other in the tissue chamber. Aβ40 only correlated with GFAP and Aβ42 only correlated weakly and negatively with total tau (Fig. 5a-b). In the circulation media, total tau levels correlated positively with NF-L, GFAP and UCH-L1 levels, and NF-L levels correlated with GFAP levels. Aβ40 levels correlated positively with total tau, NF-L and GFAP levels but not with UCH-L1 or Aβ42 levels (Fig. 5c-d). Taken together, these results support the potential utility of this model to study cerebral biomarkers and their dependence or independence to each other for mechanistic studies.

## Discussion

The interaction between neurons and the cerebrovasculature is essential for brain function and health. This neuronal-vascular interplay regulates cerebral blood flow and blood-brain exchange, and dysfunction of the NVU is associated with several neurological diseases [31]. As the lack of robust and physiologically relevant models of the human NVU is recognized to be a major roadblock for understanding the cerebrovasculature in health and disease, interest in modeling the human cerebrovasculature, particularly for capillary models, is surging. Here, we combined primary human vascular cells and human iPSC-derived neurons using a tissue engineering approach to produce a functional, human, large vessel, perfusible NVU model that can be cultured under native-like flow conditions for at least 21 days.

Early NVU models focused on modeling either vascular or neuronal components in isolation and under static conditions. Brain-derived EC, cultured alone or co-cultured with other cerebrovascular cells, often in transwells, are typically used to evaluate the ability of therapeutic agents to cross the BBB [32]. Animal- or human-derived neurons cultured in regular culture dishes lack the 3D cellular organization that regulates neuronal function and many key cellular processes in vivo. Importantly, it has become clear in the last decade that cells sense and respond to the dimensionality and rigidity of their environment, and these qualities cannot be modeled using classical tissue culture methods [33]. Newer approaches often use multicellular spheroid systems consisting of human primary or iPSC-derived EC, pericytes, astrocytes and neurons that are cultured into multicellular BBB- and/or brain-organoid structures [34–38]. These organoids can be maintained for extensive time in culture, holding great promise to study neuronal functions. However, controlled perfusion through a validated vascular lumen to study blood-to-brain and brain-to-blood transport has not yet been possible, and neurons within the center of such organoids may be nutrient starved. To address this gap, several groups have developed capillary-like NVU models using microfluidic systems. These platforms offer control over luminal flow, but focus on BBB function over neuronal function in the NVU, as the vascular cells are separated from neurons either by a porous membrane or cultured in different chips that are linked together [13, 15, 39]. Here, we opted to use bioengineering techniques to co-culture human EC, SMC, astrocytes and neurons to model the NVU structure of cortical penetrating arteries. Our approach offers the possibility to control luminal perfusion, assess bidirectional (blood-to-brain and brain-to-blood) trans-endothelial transport, as well as assess neuronal, glial and endothelial functions in a model where cell types are grown in close proximity to each other within an endogenously secreted extracellular matrix. After 21 days in culture with luminal flow, we demonstrated that abluminal neurons have histological, biochemical and electrical functions. While the measured electrical properties – in particular the resting potential – suggest that neurons in our model were not fully mature at the time of analysis, they are comparable in their electrophysiology to that previously measured in brain organoids [14]. The endothelial barrier represents an important feature of the NVU, and most previous studies use either FITC-dextran or trans endothelial electrical resistance (TEER) measurement to assess BBB integrity [40, 41]. Here we confirmed the integrity of the endothelium barrier using 4 kDa FITC-dextran. We calculated a permeability of 8.3 10E^− 9^ ± 6.06 E^− 9^ cm/s, similar to values we previously reported in bioengineered tissues composed of EC and SMC cultured in the presence (4.9 10E^− 7^ cm/s) or absence (7.0 10E^− 7^ cm/s) of astrocytes [12] or to published values (reviewed in [42]). However, dextran and TEER assays require external non-physiological chemicals to be circulated through the model, or electrical probes to be placed in the vascular lumen that could potentially lead to cellular disturbance. Thus, we also confirmed in independent tissues that astrocyte-secreted apoE remains in the tissue chamber and does not cross the endothelial barrier as documented in vivo [25] and that other fluid biomarkers show differential levels in the tissue chamber compared to the circulation media. Specifically, the differential concentrations of Aβ40, Aβ42, tau, GFAP, NF-L and UCH-L1 between the two compartments confirm tight endothelial barrier formation with specific transport mechanisms to be analyzed in future studies.

Although NVU models are being developed at a rapid pace for potential use in the study of neurodegenerative diseases, only a few studies have provided evidence supporting successful disease modeling [43, 44]. Our laboratory previously generated a vascular model composed of EC, SMC and astrocytes that can be used to study CAA, a component of AD, but the absence of neurons in our previous studies required injection of exogenous recombinant Aβ into the tissue chamber to initiate CAA [12]. Recently, Shin and colleagues developed a microfluidic AD model composed of neurons, astrocytes and EC to study Aβ-induced BBB damage [44]. Although representing an undeniable step forward in developing an in vitro model of vascular AD, this model requires the overexpression of *APP/PSEN1* genes with early onset familial AD (FAD) mutations due to short culture times, which may limit the physiologic relevance of the model to study late onset AD that accounts for the majority of total AD cases [45]. Here, we used neurons without FAD mutations and maintained engineered tissues in culture for up to 21 days to ensure Aβ secretion and deposition that closely mimics observations in the human cerebrovasculature in vivo*.* A human-based model of CAA is highly relevant, as Aβ deposition within the vascular wall is present in up to 40% of non-cognitively declined elderly brains and in 80% of late onset AD brains [46, 47]. Using this approach, we have shown that Aβ40 fibrils preferentially accumulate to a greater extent in the vasculature compared to Aβ42, in parallel to what has been shown in vivo [48, 49]. These results are concomitant to our earlier findings where we showed that recombinant Aβ42 is more amenable to lipoprotein (apoE and high-density lipoprotein)-mediated transit across and removal from the vascular wall of bioengineered cerebral vessels composed of EC and SMC with or without astrocytes than recombinant Aβ40 [12]. In our model, according to dependence calculations, the enhanced deposition of Aβ40 might not only result from an enhanced Aβ40 production in our model but also be due to an altered vascular transport or diffusion compared to Aβ42. Interestingly, Aβ40 levels are only elevated in GuHCL but not in RIPA fractions, suggesting that Aβ40 may preferentially bind to the extracellular matrix rather than accumulating intracellularly or in the interstitial fluid. Future studies are needed to fully understand the mechanisms that govern differential transport and deposition of Aβ40 and Aβ42 within the vascular wall. For example, we did not test whether tissue or media Aβ levels depend on Aβ transporter (i.e, LRP1, RAGE, glycoprotein) or Aβ binding protein (i.e. clusterin, apoE) levels. Notably, we also demonstrated that endogenous tau protein was phosphorylated in our model. Together, these results establish a promising framework to use this NVU model as an AD-relevant research tool to investigate the mechanisms of human NVU function in healthy and diseased states.

Research on fluid biomarkers for several neurological diseases is advancing at a tremendous pace, with intense interest in developing blood tests [50]. Here we demonstrated that several neuronal and glial biomarkers used in clinical studies could be detected in both the tissue chamber and circulation media. Although the absolute biomarker levels were different compared to in vivo conditions, the ratio of circulation:chamber levels was comparable to human blood: CSF ratios [51–55]. Thus, our model offers the potential to study how specific protein levels correlate in “brain” to “blood” compartments to understand the dependence and independence among biomarkers, recognizing that our model lacks peripheral catabolic pathways that govern clearance and excretion of blood proteins from the body. Using this approach, we found that in the tissue chamber, tau and NF-L levels do not correlate with Aβ40 levels, but the levels of these biomarkers do correlate in the circulation media. This suggests that blood tau and NF-L levels are independent of the amount of Aβ40 produced, but appear to depend on the amount of Aβ40 retained in the vascular wall. Future studies are required to fully define these relationships and compare them to in vivo data. Of particular interest would be measures of NF-L and tau levels in CAA patients. Importantly, the correlation analyses presented here only represents the statistical dependence between two variables and further studies are required to fully define the relationships and compare them to in vivo data.

This study has several limitations. The stiffness of the current scaffold material used in this model prevents studying vascular compliance, which is an important outcome measure of neurovascular coupling. Refinement of a more suitable scaffold material may enable the study of SMC contraction and relaxation in response to neuronal activity or circulating stimuli, which is an important objective given that several neurodegenerative disease patients have altered CBF [8, 56, 57] and CBF regulation happens both in arterioles and in capillaries [7]. Another limitation is that this study used cells derived from umbilical cord, due to the slow growth rate and expense of primary human cerebrovascular cells. Although cord cells clearly do not reflect the physiology of brain vasculature, we and others have previously demonstrated that HUVECs express BBB marker proteins when co-cultured with astrocytes and thus may be suitable as a cost-effective cellular source for model development [12, 58, 59]. That apoE, which does not cross the BBB [25], is restricted to the abluminal chamber in our model supports the conclusion that HUVEC can form a functional endothelial barrier. The choice to use primary cells over iPSC-derived cells was motivated by the accessibility of primary human EC, SMC and astrocytes, and because we previously reported the feasibility to bioengineer cerebral vessels using these cells [12, 19]. Future studies will be required to develop tissues made entirely from patient-derived iPSCs for personalized medicine applications. Importantly, as iPSCs will be specific to each individual donor, they have the potential to be highly variable between donor batches, and studies using isogenic iPSC sets are encouraged. Another limitation is that the protocol used here for iPSC neuronal differentiation is known to generate a mixed population of cells positive for the neuronal markers MAP 2 and β-tubulin-III (> 75%) and cells positive for GFAP (< 25%), as previously reported [20]. Despite these limitations, our study is a proof-of-concept demonstration that a cerebral arterial NVU can be engineered in vitro as a potentially relevant tool to study neurodegenerative diseases.

## Conclusion

Our arterial NVU model represents an important step toward the development of a human-based translational in vitro model of large cerebral vessels. It could be a useful research tool for the study of cerebrovascular function in both physiological and pathophysiological conditions. In particular, as our model offers the possibility to study transport from the brain to the circulation and vice versa, it also has the potential to be a novel and relevant platform for drug development targeting both neuronal and vascular function, and opens up the possibility to study neuronal and glial biomarkers.

## Supplementary Information

**Additional file 1: Supplemental Figure 1.** Histological structure of human arterial NVU. **a**) Human cortex (Brodmann area 9) were stained against **a**) vWF (EC marker), αSMA (SMC marker) and GFAP (astrocyte marker) and against **b**) MAP 2 (neuron marker), αSMA (SMC marker) and GFAP (astrocyte marker) to visualize the cerebrovasculature.

**Additional file 2: Supplemental Figure 2.** Histological characterization and function of bioengineered arterial NVU. **a**) The histological structure of the arterial NVU was assessed by H&E staining. b) The astrocytes in arterial NVU were further analyzed with immunostaining against aquaporin 4 (AQ4). Barrier integrity was assessed by measuring permeability of 250 μg/ml of 4 kDa FITC-dextran circulated through the lumen for 2 h. Points in graphed data represent individual bioengineered vessels, bars represent mean, error bars represent ±SEM. ABL = antelumen.

**Additional file 3: Supplemental Figure 3.** Aβ40 and Aβ42 concentration in circulation media. (**a**) Aβ40 and (**b**) Aβ42 levels in circulation media of tissue composed of EC and SCM (bipartite), EC, SMC and astrocyte (tripartite) and EC, SMC, astrocytes and neurons (NVU) after 1 or 3 weeks in culture were quantified by ELISA. Points in graphed data represent individual bioengineered vessels, bars represent mean, error bars represent ±SEM and analysed by one way ANOVA. Values below the detection of the ELISA are plotted in gray. * = *p* < 0.05, ** = *p* < 0.01, *** = *p* < 0.001.

**Additional file 4: Supplemental Figure 4.** Aβ and p-tau histology. Cryopreserved bioengineered arterial NVU were cut longitudinally to show a cross-section of the NVU wall. **a)** Immunohistochemistry against OC fibril confirmed the deposition of Aβ fibril both in the neuron layer (β-tub III positive, white arrow) as well as deeper in the vascular wall (blue arrows). **b**) The expression of Aβ was further investigated by staining against Aβ 1–16. Positive signal was found both deep in the vascular wall (white arrow) as well as co-localized within MAP 2 positive cells (yellow arrow). **c**) Immunohistochemistry against AT8 suggest the deposition of p-tau both within neuron (MAP 2 positive, yellow arrow) as well as extracellular (white arrow), purple arrow shows the remaining of the scaffold. Human brains were used as representative staining pattern. ABL = albumen.

## Data Availability

Raw data can be obtained from corresponding authors.
