# Supplementary figures and images for: An in vitro bioengineered model of the human arterial neurovascular unit to study neurodegenerative diseases
Source: Mol Neurodegener. 2020 Nov 19;15:70. doi: 10.1186/s13024-020-00418-z (PMC7678181; doi:10.1186/s13024-020-00418-z)

Sup. Fig. 1

a

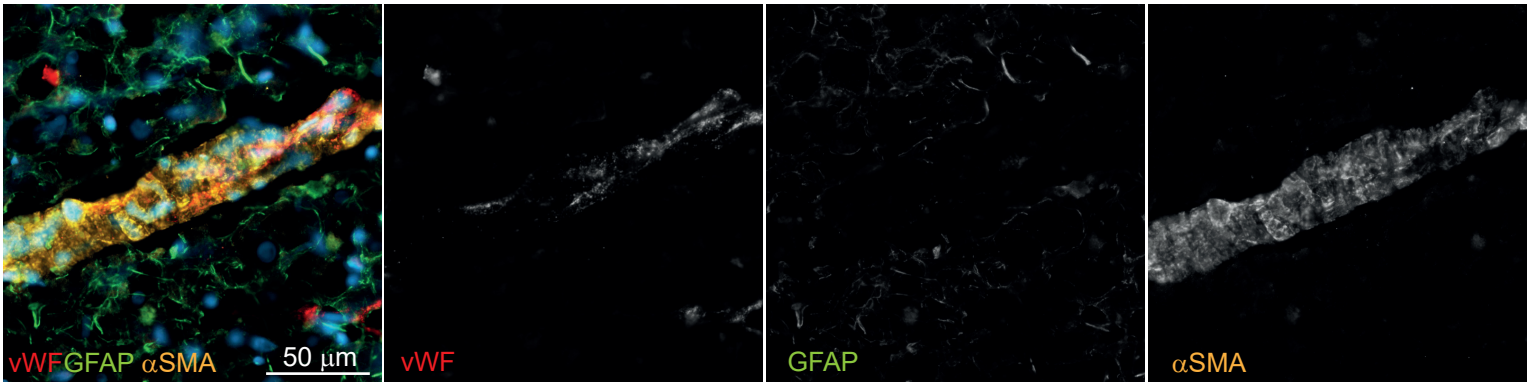

b

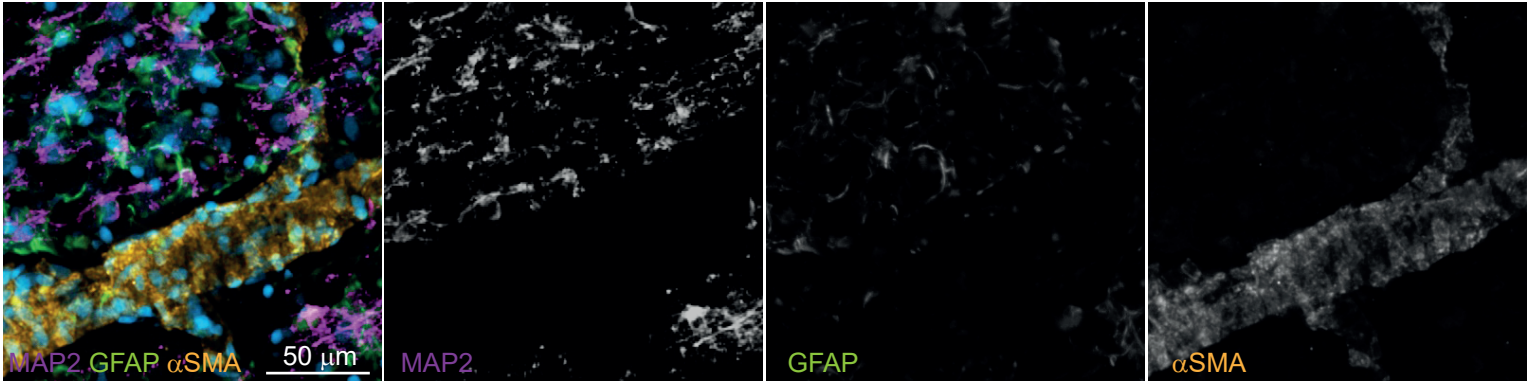

Supplement: Supplementary file 1 — Additional file 1: Supplemental Figure 1. Histological structure of human arterial NVU. a) Human cortex (Brodmann area 9) were stained against a) vWF (EC marker), αSMA (SMC marker) and GFAP (astrocyte marker) and against b) MAP 2 (neuron marker), αSMA (SMC marker) and GFAP (astrocyte marker) to visualize the cerebrovasculature. [file 13024_2020_418_MOESM1_ESM.pdf]

Sup. Fig. 2

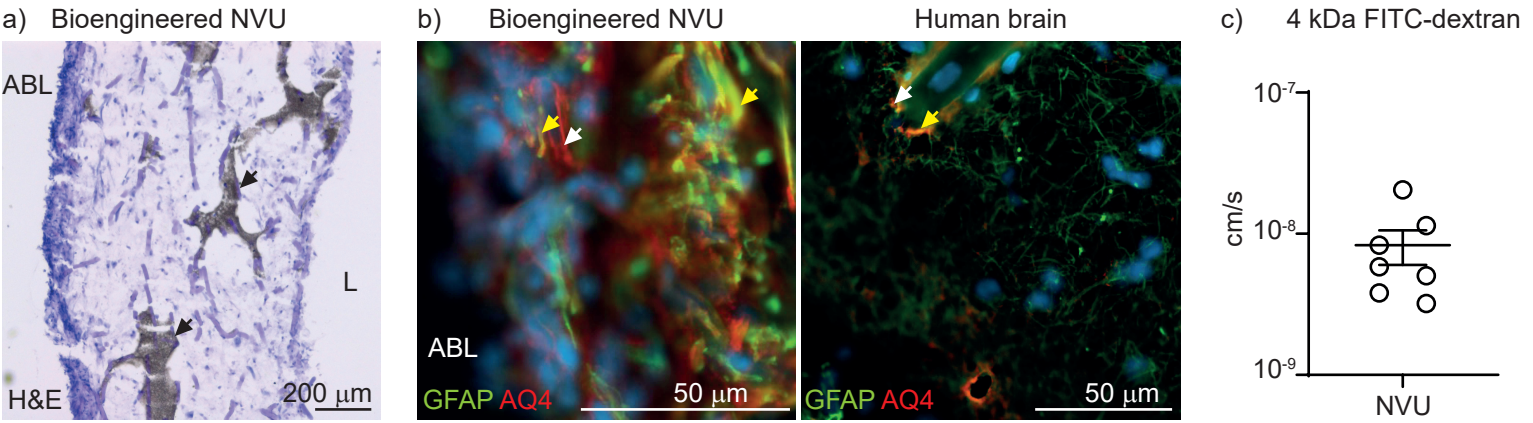

Supplement: Supplementary file 2 — Additional file 2: Supplemental Figure 2. Histological characterization and function of bioengineered arterial NVU. a) The histological structure of the arterial NVU was assessed by H&E staining. b) The astrocytes in arterial NVU were further analyzed with immunostaining against aquaporin 4 (AQ4). Barrier integrity was assessed by measuring permeability of 250 μg/ml of 4 kDa FITC-dextran circulated through the lumen for 2 h. Points in graphed data represent individual bioengineered vessels, bars represent mean, error bars represent ±SEM. ABL = antelumen. [file 13024_2020_418_MOESM2_ESM.pdf]

Sup. Fig. 3

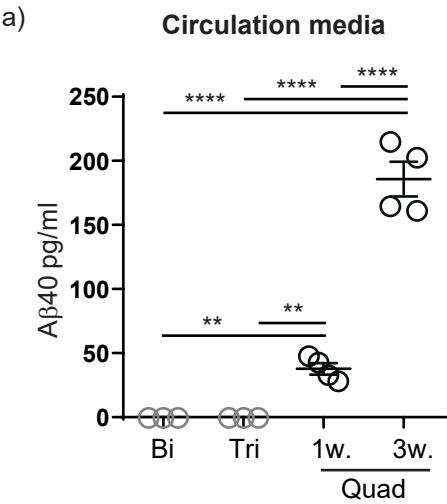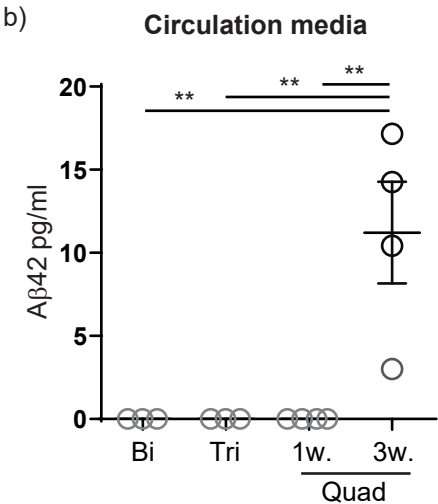

Supplement: Supplementary file 3 — Additional file 3: Supplemental Figure 3. Aβ40 and Aβ42 concentration in circulation media. (a) Aβ40 and (b) Aβ42 levels in circulation media of tissue composed of EC and SCM (bipartite), EC, SMC and astrocyte (tripartite) and EC, SMC, astrocytes and neurons (NVU) after 1 or 3 weeks in culture were quantified by ELISA. Points in graphed data represent individual bioengineered vessels, bars represent mean, error bars represent ±SEM and analysed by one way ANOVA. Values below the detection of the ELISA are plotted in gray. * = p < 0.05, ** = p < 0.01, *** = p < 0.001. [file 13024_2020_418_MOESM3_ESM.pdf]
