# Supplementary material for: An in vitro bioengineered model of the human arterial neurovascular unit to study neurodegenerative diseases
Source: Mol Neurodegener. 2020 Nov 19;15:70. doi: 10.1186/s13024-020-00418-z (PMC7678181; doi:10.1186/s13024-020-00418-z)

Sup. Fig. 4

a) Aβ fibrils

Bioengineered NVU | Human brain

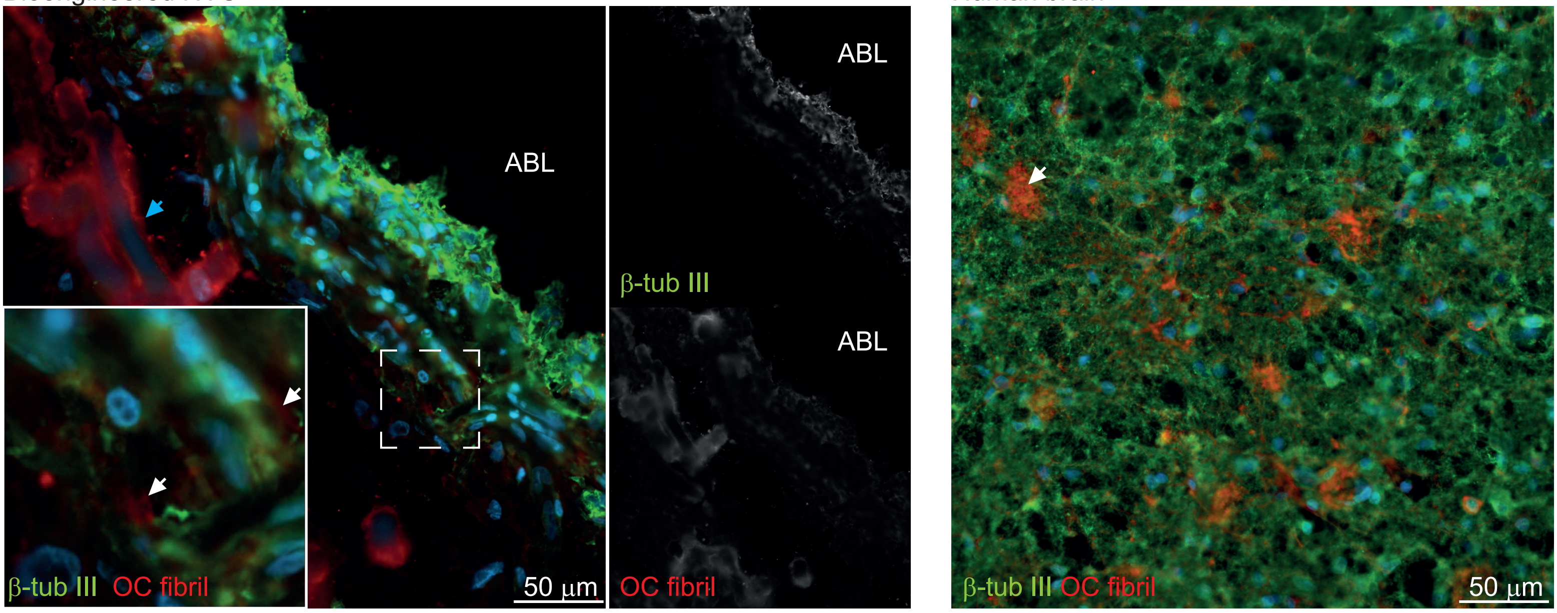

b) Aβ 1-16

Bioengineered NVU | Human brain

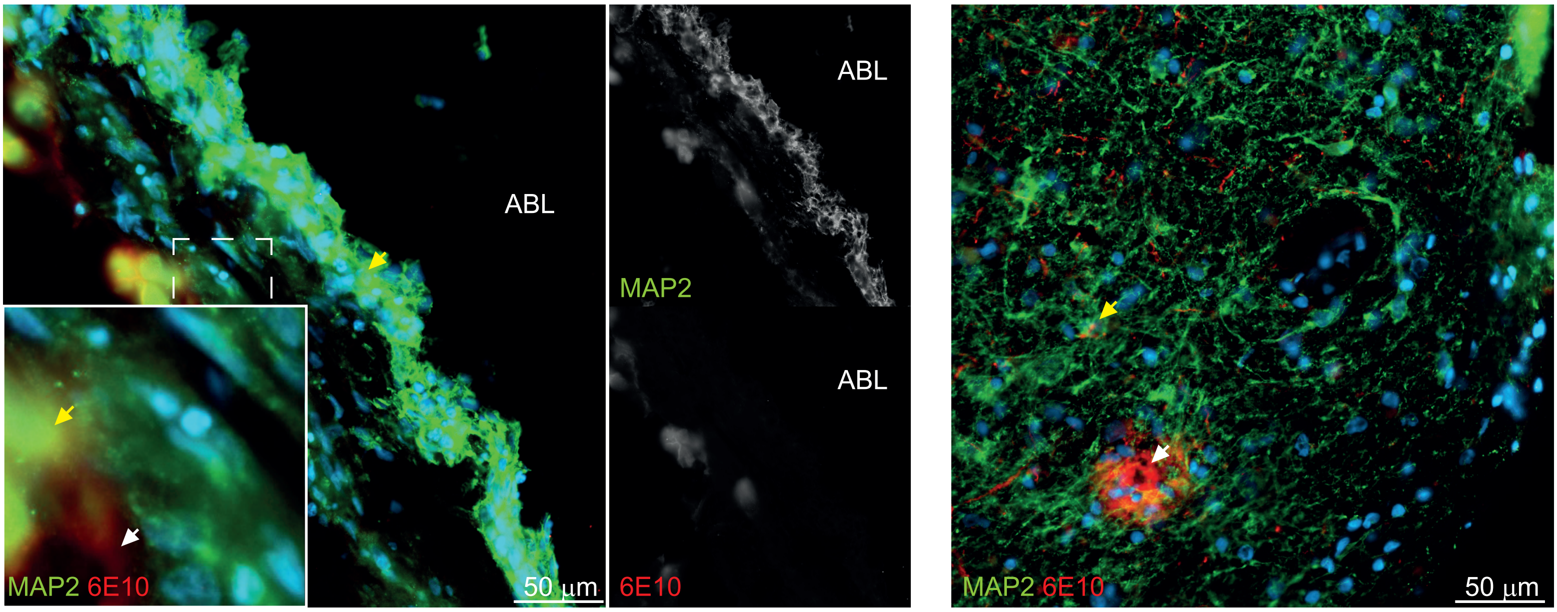

c) p-Tau

Bioengineered NVU | Human brain

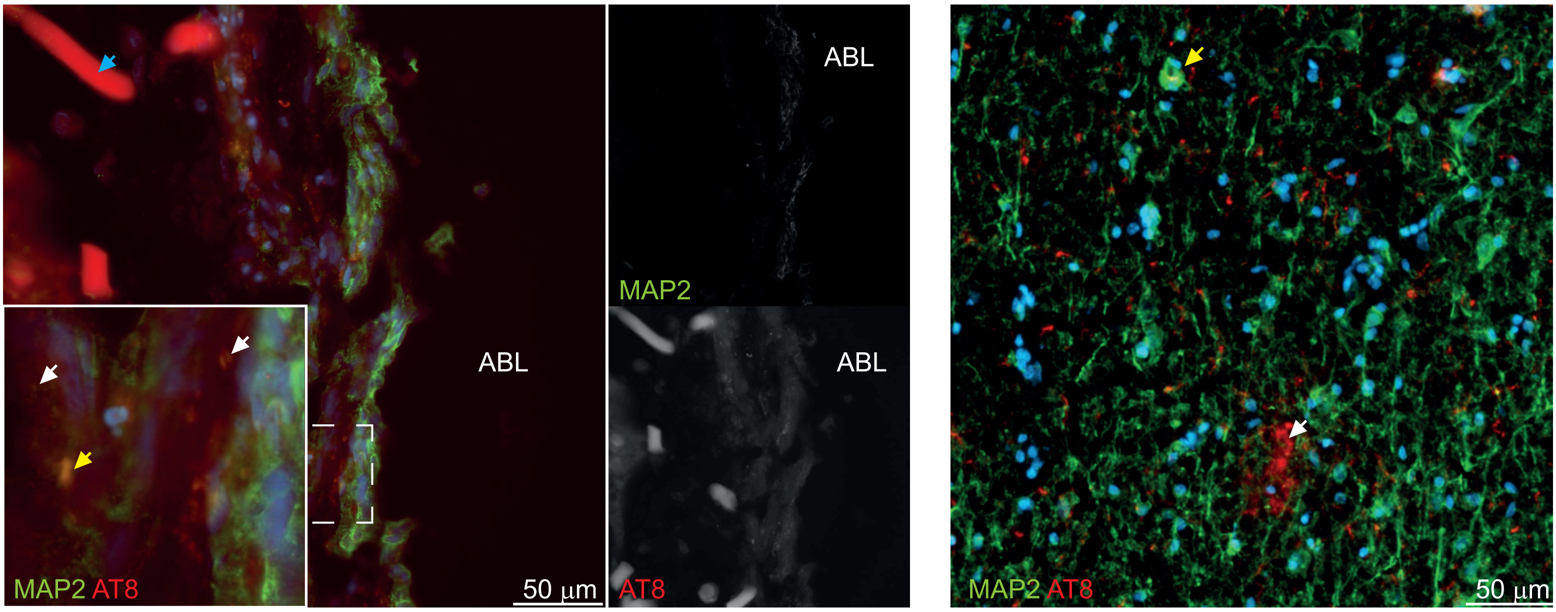

Supplement: Supplementary file 4 — Additional file 4: Supplemental Figure 4. Aβ and p-tau histology. Cryopreserved bioengineered arterial NVU were cut longitudinally to show a cross-section of the NVU wall. a) Immunohistochemistry against OC fibril confirmed the deposition of Aβ fibril both in the neuron layer (β-tub III positive, white arrow) as well as deeper in the vascular wall (blue arrows). b) The expression of Aβ was further investigated by staining against Aβ 1–16. Positive signal was found both deep in the vascular wall (white arrow) as well as co-localized within MAP 2 positive cells (yellow arrow). c) Immunohistochemistry against AT8 suggest the deposition of p-tau both within neuron (MAP 2 positive, yellow arrow) as well as extracellular (white arrow), purple arrow shows the remaining of the scaffold. Human brains were used as representative staining pattern. ABL = albumen. [file 13024_2020_418_MOESM4_ESM.pdf]
